# Comparative Feeding Ecology of Bull Sharks (*Carcharhinus leucas*) in the Coastal Waters of the Southwest Indian Ocean Inferred from Stable Isotope Analysis

**DOI:** 10.1371/journal.pone.0078229

**Published:** 2013-10-21

**Authors:** Ryan Daly, Pierre W. Froneman, Malcolm J. Smale

**Affiliations:** 1 Department of Zoology & Entomology, Rhodes University, Grahamstown, Eastern Cape, South Africa; 2 Port Elizabeth Museum at Bayworld, Port Elizabeth, Eastern Cape, South Africa; 3 Department of Zoology, Nelson Mandela Metropolitan University, Port Elizabeth, Eastern Cape, South Africa; University of California Davis, United States of America

## Abstract

As apex predators, sharks play an important role shaping their respective marine communities through predation and associated risk effects. Understanding the predatory dynamics of sharks within communities is, therefore, necessary to establish effective ecologically based conservation strategies. We employed non-lethal sampling methods to investigate the feeding ecology of bull sharks (*Carcharhinus leucas*) using stable isotope analysis within a subtropical marine community in the southwest Indian Ocean. The main objectives of this study were to investigate and compare the predatory role that sub-adult and adult bull sharks play within a top predatory teleost fish community. Bull sharks had significantly broader niche widths compared to top predatory teleost assemblages with a wide and relatively enriched range of δ^13^C values relative to the local marine community. This suggests that bull sharks forage from a more diverse range of δ^13^C sources over a wider geographical range than the predatory teleost community. Adult bull sharks appeared to exhibit a shift towards consistently higher trophic level prey from an expanded foraging range compared to sub-adults, possibly due to increased mobility linked with size. Although predatory teleost fish are also capable of substantial migrations, bull sharks may have the ability to exploit a more diverse range of habitats and appeared to prey on a wider diversity of larger prey. This suggests that bull sharks play an important predatory role within their respective marine communities and adult sharks in particular may shape and link ecological processes of a variety of marine communities over a broad range.

## Introduction

Large shark species are often top predators within their respective marine ecosystems and consequently, play an important role shaping community dynamics [1]. As habitat loss and over-fishing has increasingly lead to the decline of shark populations [2,3], the ecological consequences of their removal from marine communities have been substantial [4,5]. Sharks may shape their communities through direct predation or associated risk effects [6] but the understanding behind broad spatial and temporal scales over which these processes operate is poor. Often it is difficult to conduct long-term studies on large sharks throughout their range because they are rare, difficult to observe, highly mobile and often widespread. The application of stable isotope analysis (SIA) applied to investigate the trophic ecology of large shark species has, however, provided new insights into the processes through which they shape their communities [7]. 

Traditionally, investigating the trophic ecology of large sharks has relied on stomach content analysis from captured dead sharks [8,9]. The limitations of this method arise from the snapshot nature of recently consumed dietary items, the logistical difficulties associated with sampling, and conservation related concerns of sampling threatened populations [10]. Although SIA may not provide the taxonomic resolution of stomach content analyses, it does provide information on the assimilated diet of the consumer over time [11]. Refined by recent progress in the application of stable carbon and nitrogen isotope analysis to study elasmobranchs [12-15], the associated methodology is a robust and complementary tool for investigating the trophic ecology of elasmobranchs. The application of SIA provides insight into various population, species and individual level processes including the trophic position [11,16], niche width and overlap [17], ontogenetic dietary shifts [18,19], species foraging strategies [20] individual foraging strategies [19,21], and habitat use [22-24] of large sharks.

The bull shark, *Carcharhinus leucas*, is a large apex predator distributed throughout the coastal regions of the tropical and warm temperate oceans [25]. Juveniles occur in estuarine nursery areas that are typically accessed more easily than the coastal marine environment and have been the focus of many previous studies [26-30]. Less is known about the habitat use and trophic ecology of sub-adult and adult populations in the south west Indian Ocean, specifically in Mozambique where studying these populations remains logistically challenging. The diet of bull sharks typically includes a wide variety of prey items [31] but the spatial and temporal scales over which individuals forage is unclear. Bull sharks are known to exhibit periods of extended site fidelity [31,32] that may be linked with resource availability but individual home ranges may vary over broad spatial and temporal scales [33,34]. Bull sharks are capable of ranging over wide geographical areas [33,34] and undertake seasonal migrations [25,26,35] but little is known about how bull shark foraging behavior links with these processes. There is also uncertainty related to how foraging behavior may vary within a population or between individuals. Recent studies suggest that juvenile bull sharks exhibit some level of individual dietary specialization [21], however there is little knowledge regarding individual dietary specialization of larger sharks and how factors such as ontogeny, gender or habitat use affect the trophic dynamics of individuals or populations. 

This study employed SIA to investigate the trophic ecology of bull sharks within a coastal marine community. The aims of this investigation were (1) to investigate the isotopic composition of the sampled marine community at the study site (2), to investigate potential bull shark dietary sources at the study site (3), to determine and compare the niche width of sub-adult and adult bull sharks and co-occurring predatory teleost fish assemblages and (4) to calculate the δ^15^N based trophic position of the sampled bull shark population. As bull sharks are especially vulnerable to increasing pressure from overfishing and habitat loss globally [2] due to their affinity to coastal habitats and low intrinsic rebound potential [36], information on their predatory role within the sampled marine community may help to establish a more effective conservation strategy [37]. 

## Methods

### Ethics Statement

All research in this investigation was conducted under the permit number 0002/2010 issued by The Mozambican Directorate of National Conservation Areas. The Animal Ethics Committee of the Department of Zoology and Entomology at Rhodes University approved the research protocol used in this study (ethical clearance number ZOOL-14-2012). 

### Study site

**Figure 1:**
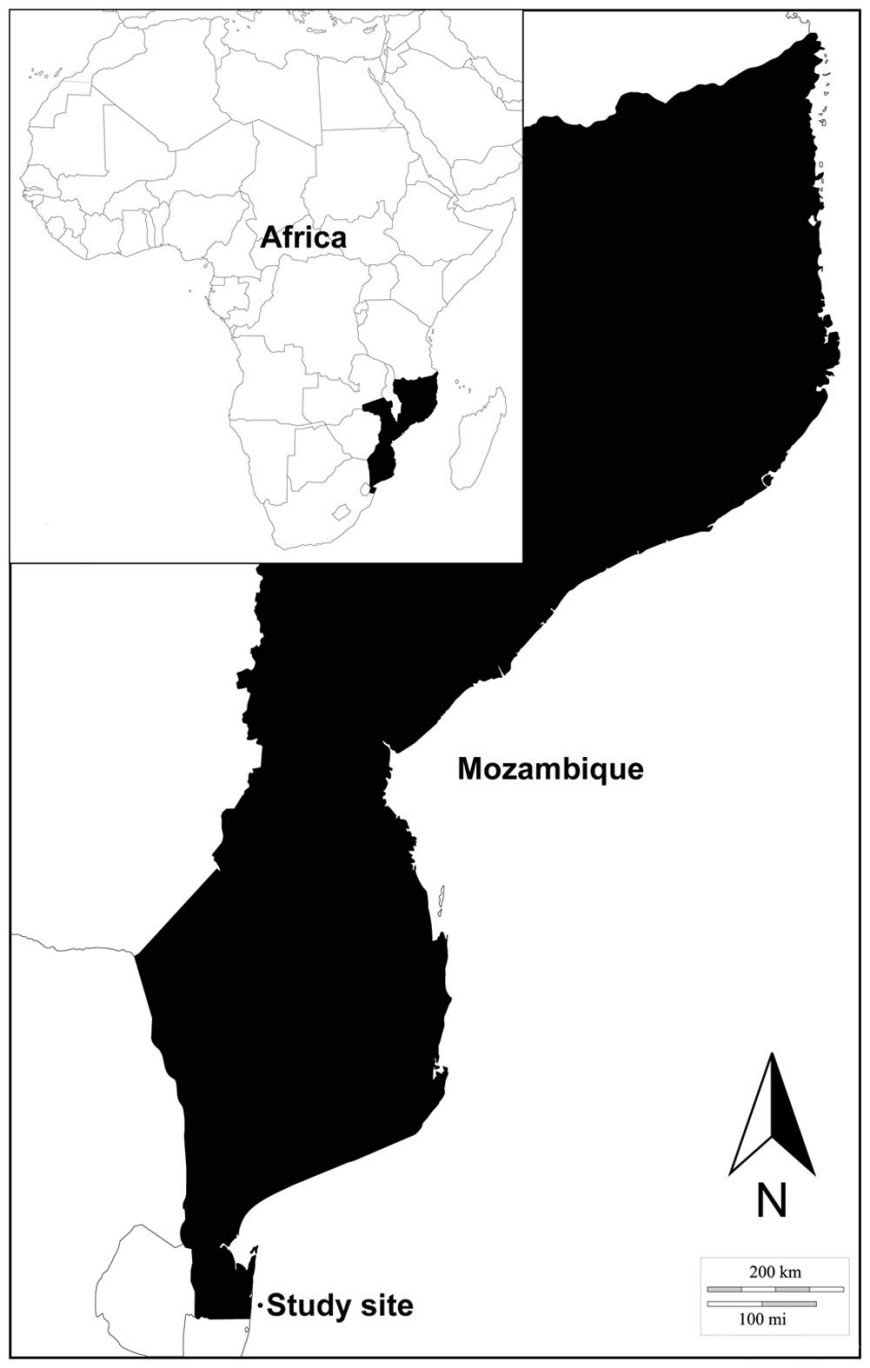
The study site is located in the southwest Indian Ocean off the coast of southern Mozambique within the Ponta do Ouro Partial Marine Reserve.

This study took place in southern Mozambique (S26° 44.934’ E32° 56.083’) approximately 12km north of the South African border (Figure 1). The marine environment is a transition zone between the temperate southern African and tropical western Indo-Pacific marine ecoregions [38] and has some of the world’s highest latitude hard coral reefs [39] and a diverse Indo-Pacific fish community [40]. Sampling was conducted within the Ponta do Ouro Partial Marine Reserve and focused on a reef complex called the pinnacles. The pinnacles reef is situated approximately 3.7km offshore and forms a shallow ridge (30m) surrounded by deeper water (50m+). During austral summer this reef is an aggregation site for bull sharks as well as a diversity of predatory teleost species (pers obs.). Individual bull shark site fidelity at the study site may be variable but some sharks do exhibit high levels of site fidelity during summer months (December to May) and may return to the study site for multiple years. 

### Sample collection and preperation

Visual assessments of the fish community took place over the course of 111 dives at the study site. Based on these observations, samples from predatory fish species were obtained by selectively spear fishing the most numerically abundant species. Other fish species sampled were primarily obtained from artisanal fishermen at the study site and opportunistic biopsy samples were obtained from blacktip sharks (*Carcharhinus limbatus*) at the study site (Table 1). Sampling effort was focused on austral summer months (November to April) between January 2009 and March 2012. A total of 59 samples from 16 teleost fish species and two small shark species were collected and white muscle tissue was used for stable isotope analysis. Fish species were grouped into categories based on primary habitat type, trophic position and primary prey items known from the literature (Table 1) [41-47].

**Table 1:** Sampled teleost fish and shark species assigned to four groups based on their primary habitat, primary dietary items and trophic position (TP) based on stomach content established from www.fishbase.org.

**Species**	**Common name (n)**	**TP (SE)**	**Primary Habitat**	**Primary Diet**	**Assemblage**
**Group 1**					
*Acanthocybium solandri*	Wahoo (8)	4.4 (0.8)	Offshore / Pelagic	Fish / Invertebrates	Pelagic Top Predator
*Coryphaena hippurus*	Dorado (4)	4.4 (0.8)			
*Istiophorus platypterus*	Sailfish (1)	4.5 (0.8)			
*Euthynnus affinis*	Kawakawa (1)	4.5 (0.8)			
**Group 2**					
*Scomberomorus commerson*	King mackerel (10)	4.5 (0.8)	Coastal / Pelagic	Fish / Invertebrates	Coastal Top Predator
*Carangoides fulvoguttatus*	Yellowspotted trevally (6)	4.4 (0.8)			
*Seriola dumerili*	Greater amberjack (3)	4.5 (0.8)			
*Caranx sexfasciatus*	Bigeye trevally (4)	4.5 (0.8)			
*Sphyraena jello*	Pickhandle barracuda (4)	4.5 (0.8)			
**Group 3**					
*Lutjanus gibbus*	Humpback snapper (3)	3.6 (0.6)	Coastal / Benthic	Invertebrates	Coastal Consumer
*Plectorhinchus playfairi*	Rubber lips (4)	3.3 (0.52)			
*Umbrina robinsoni*	Slender baardman (1)	3.4 (0.42)			
*Balistapus undulates*	Orange-striped trigger fish (1)	3.4 (0.42)			
*Rhabdosargus sarba*	Natal stumpnose (1)	3.4 (0.6)			
*Chrysoblephus Puniceus*	Slinger (1)	3.5 (0.46)			
*Oplegnathus robinsoni*	Natal knifejaw (1)	3.2 (0.42)			
**Group 4**					
*Carcharhinus limbatus*	Blacktip shark (4)	4.2 (0.7)	Coastal / Pelagic	Fish / Generalist	Elasmobranch
*Rhizoprionodon acutus*	Milk shark (2)	4.3 (0.8)			

Shark muscle tissue samples for stable isotope analysis were obtained from free-swimming sharks using a biopsy probe attached to the tip of an underwater spear gun [48]. Samples from 18 bull sharks were obtained and the respective gender was identified *in situ* when possible and confirmed from video footage. The presence of claspers confirmed male sharks but in some cases the gender of sub-adult sharks was not clear due to the smaller size of the claspers and in total only 11 individuals were positively sexed. All sampled sharks were measured *in situ* using laser photogrammetry [48] and ranged in size from 1.6 - 2.5m TL (2m ±0.3, mean ±SD). Bull sharks were then grouped into sub-adults (1.6m-2.2m) and adults (2.2m-2.5 TL) based on the approximate length at sexual maturity (c. 2.2m TL) according to the literature [26,49-51]. 

All tissue samples used for stable isotope analysis were frozen at -20°C before transport to Rhodes University, South Africa. In the laboratory samples were oven dried at 60°C for 48 hours or until constant weight was reached and were then homogenized into a fine powder using a Crescent Wig-L-Bug. Samples were then weighed to approximately 1mg using a Sartorius micro-balance with a precision of 0.01mg and placed in 6x4mm tin capsules before being sent to Iso-Environmental at Rhodes University in South Africa. Samples were analyzed using a continuous flow Europa Scientific 20-20 IRMS linked to an ANCA SL Prep Unit. Each batch of 96 combustions contained 29 internal standards (beet sugar and ammonium sulphate) and 5 certified protein standards (Casein calibrated against IAEA-CH-6 and IAEA-N-1). Stable isotope ratios were expressed in the delta notation where δ^13^C or δ^15^N = [(R_sample_/R_standard_)-1] × 1000 and R is ^13^C/^12^C or ^15^N/^14^N and nitrogen, δ^15^N. 

As the presence of lipids in the muscle tissue samples may lead to depleted δ^13^C values [7,52], samples were checked for skewed C:N ratios. All samples had low C:N ratios (3.28 ± 0.31, mean ± SD) which were not correlated with δ^13^C (R^2^=0.26) suggesting that lipid content in the samples was negligible [52]. Bull shark samples specifically had a low C:N ratio (3.1 ± 0.18, mean ± SD) confirming that lipid content was low. The presence of urea in elasmobranch tissue may also lead to depleted δ^15^N values [7,14] but for the purposes of this investigation it was not accounted for due to the variable and possibly negligible effects on elasmobranch muscle tissues [13]. 

### Data analysis

Differences in mean and variance of the carbon and nitrogen isotope values of bull sharks between males and females and adults and sub-adults were investigated. The mean difference and variance in carbon and nitrogen isotope values between bull sharks and fish groups 1-3 were also calculated. In all cases the data were tested for normality using a Shapiro-Wilk test and square root transformed where applicable. To test for differences between groups a t-test for independent samples (for normally distributed data) or a Wilcoxon Signed Rank test (for non-normally distributed data) was used. To investigate variance between groups a Bartletts test for homogeneity of variances was performed in the statistical package R (CRAN 2009). A linear regression analysis was also conducted to investigate the relationship between shark size (TL) and δ^13^C and δ^15^N values. 

Niche widths and overlap for bull sharks and fish groups 1 and 2 were calculated using SIBER (Stable Isotope Bayesian Ellipses in R) metrics [54] in the R statistics platform (CRAN 2009). Small sample size corrected Bayesian ellipses were employed to account for potential bias between sample sizes when performing comparative analysis between groups. The area of the small sample size corrected ellipses was used to represent niche width. To compare differences in niche width between groups, the proportion of Bayesian ellipses (initially calculated using the model in the SIBER package) that were larger or smaller relative to the compared group were calculated and represented as a probability value between 1 and 100. The relative contribution of different fish groups to the diet of bull sharks was estimated using the isotope mixing model SIAR (stable isotope analysis in R) [55] in the R statistics platform (CRAN 2009). The model was run using sub-adult and adult bull sharks as the consumers and teleost fish groups 1-3 and shark group 4 were used as sources. Although fish groups 1 and 2 were grouped separately *a priori* due to known differences between habitat preference and primary prey items, they exhibited no significant differences between δ^13^C and δ^15^N values. In order not to confound the mixing model, groups 1 and 2 were combined for this analysis. Trophic fractionation values for bull sharks used to run the model were 2.29‰, ±0.22 (mean, ±SD) for Δ15N and 0.90‰, ±0.33 (mean, ±SD) for Δ13C [12]. Concentration factors were not incorporated into the model because the variation between the source values of carbon and nitrogen isotopes was negligible [56,57]. The results of the mixing model showing the calculated bull shark dietary proportions were represented as box plots indicating the 25, 75 and 95% confidence intervals. 

To determine the trophic position of bull sharks we used the equation TP = λ + (δ^15^N_consumer_ - δ^15^N_base_) / Δ_n_, where λ is the trophic position of the consumer used as a baseline, δ^15^N_base_ is the mean δ^15^N of this baseline, δ^15^N_consumer_ is the δ^15^N value of bull sharks and Δ_n_ was the fractionation value of the consumer [53]. The value chosen for the base consumer was the mean nitrogen isotope value of fishes from group 3 (11.01‰) as this group accounted for a high proportion of potential dietary items (as calculated by the mixing model) with a mean trophic position of 3.4 (Table 1). Trophic positions for each fish species were obtained from www.fishbase.org [47] and were based on analyses performed on stomach contents of the respective fishes. The fractionation value used for muscle tissue of δ^15^N for bull sharks was 2.29 [12]. 

## Results

### Community isotopic composition

**Figure 2:**
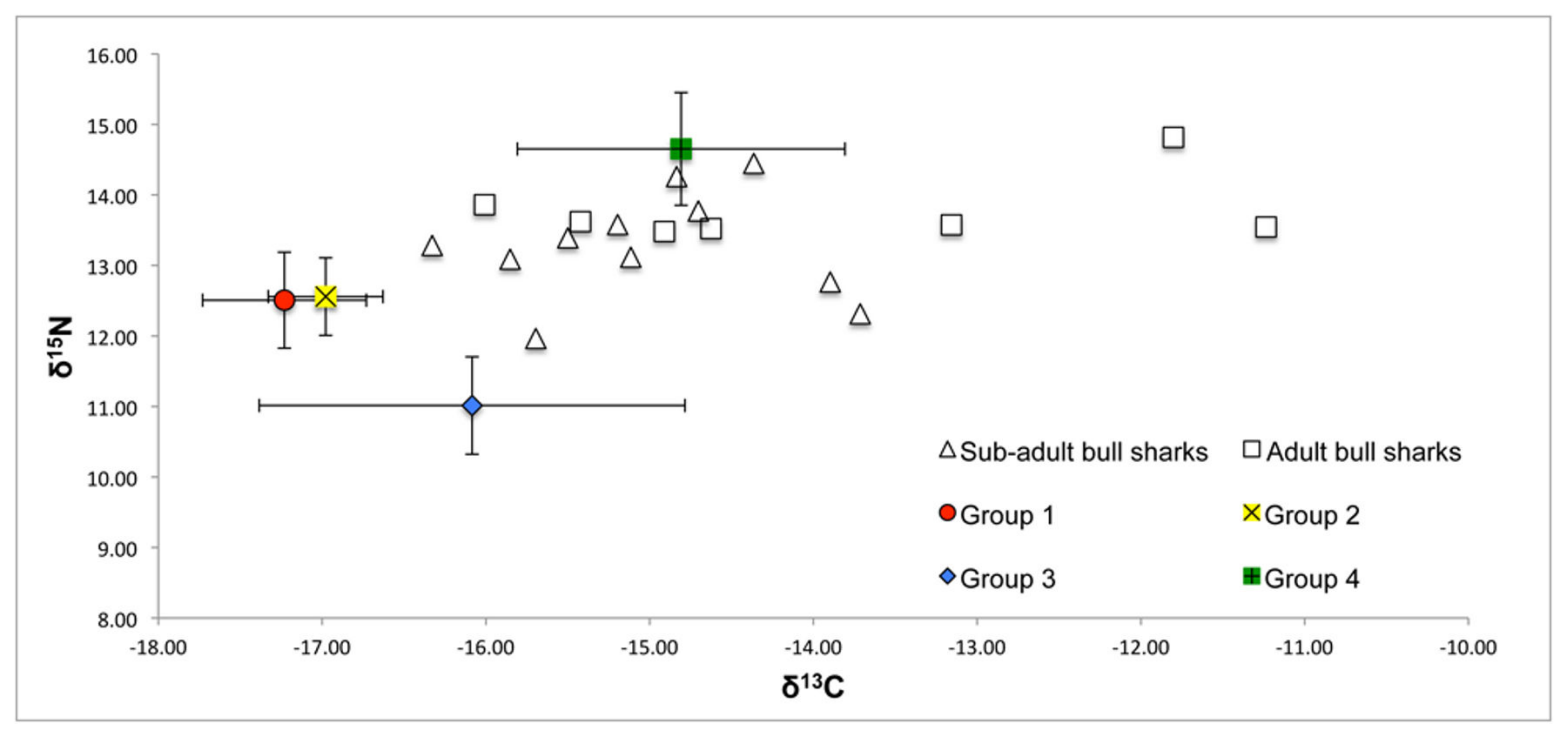
A dual isotope plot representing the sampled fish community and individual bull sharks. Fish groups represented by the mean δ^13^C and δ^15^N values (±SD). Adult individual bull sharks are represented by squares and sub-adults are represented by triangles.

Figure 2 shows the results from the stable isotope analysis plotted in isotopic niche space representing the mean (±SD) δ^13^C and δ^15^N values of groups 1-4 and individual bull shark samples representing sub-adult and adult sharks. Fish groups 1 and 2 exhibited similar mean δ^15^N values and group 2 had slightly more enriched mean δ^13^C values with no significant (p>0.05) difference in variance between the δ^15^N and δ^13^C values of these groups (Table 2). Fish group 3 had relatively depleted δ^15^N values but a significantly greater variance (p<0.05) of more enriched δ^13^C values relative to groups 1 and 2 (Table 2). Elasmobranch group 4 exhibited relatively enriched δ^13^C and δ^15^N values relative to groups 1-3 with δ^13^C values within the same range as those exhibited by bull sharks with a more enriched δ^15^N mean value (Table 2). 

**Table 2:** A summary of the mean δ^15^N and δ^13^C values for all groups and calculated niche widths of adult and sub-adult bull sharks and fish groups 1 and 2.

**Group**	**Mean δ^15^N (‰) (Range)**	**Mean δ^13^C (‰) (Range)**	**Niche Width**
All bull sharks	13.5 (2.9)	-14.6 (5.11)	3.1
Adult bull sharks	13.8 (1.3)	-13.9 (4.8)	3.1
Sub-adult bull sharks	13.3 (2.5)	-15.0 (2.6)	2.1
Group 1	12.5 (1.1)	-17.2 (2.2)	1.1
Group 2	12.6 (4.3)	-17.0 (2.1)	0.9
Group 3	11.0 (2.1)	-16.1 (4.4)	-
Group 4	14.65 (0.8)	-14.81 (1.0)	-

### Bull shark stable isotope analysis

No significant differences in δ^13^C and δ^15^N between male and female bull sharks were apparent (p > 0.05). Additionally, there was no significant relationship between δ^13^C (R^2^ = 0.2, p>0.05) and δ^15^N values (R^2^ = 0.01, p>0.05) and shark size. However, adult sharks did exhibit significantly greater variance (p<0.05) of more enriched δ^13^C values (range = 4.8‰) compared with sub-adult values (range = 2.6‰). By contrast, the δ^15^N values did not exhibit significant variance (p>0.05) but were narrower and more enriched in adult (range = 1.3‰) compared to sub-adult sharks (range = 2.5‰) (Table 2). 

### Mixing model

**Figure 3:**
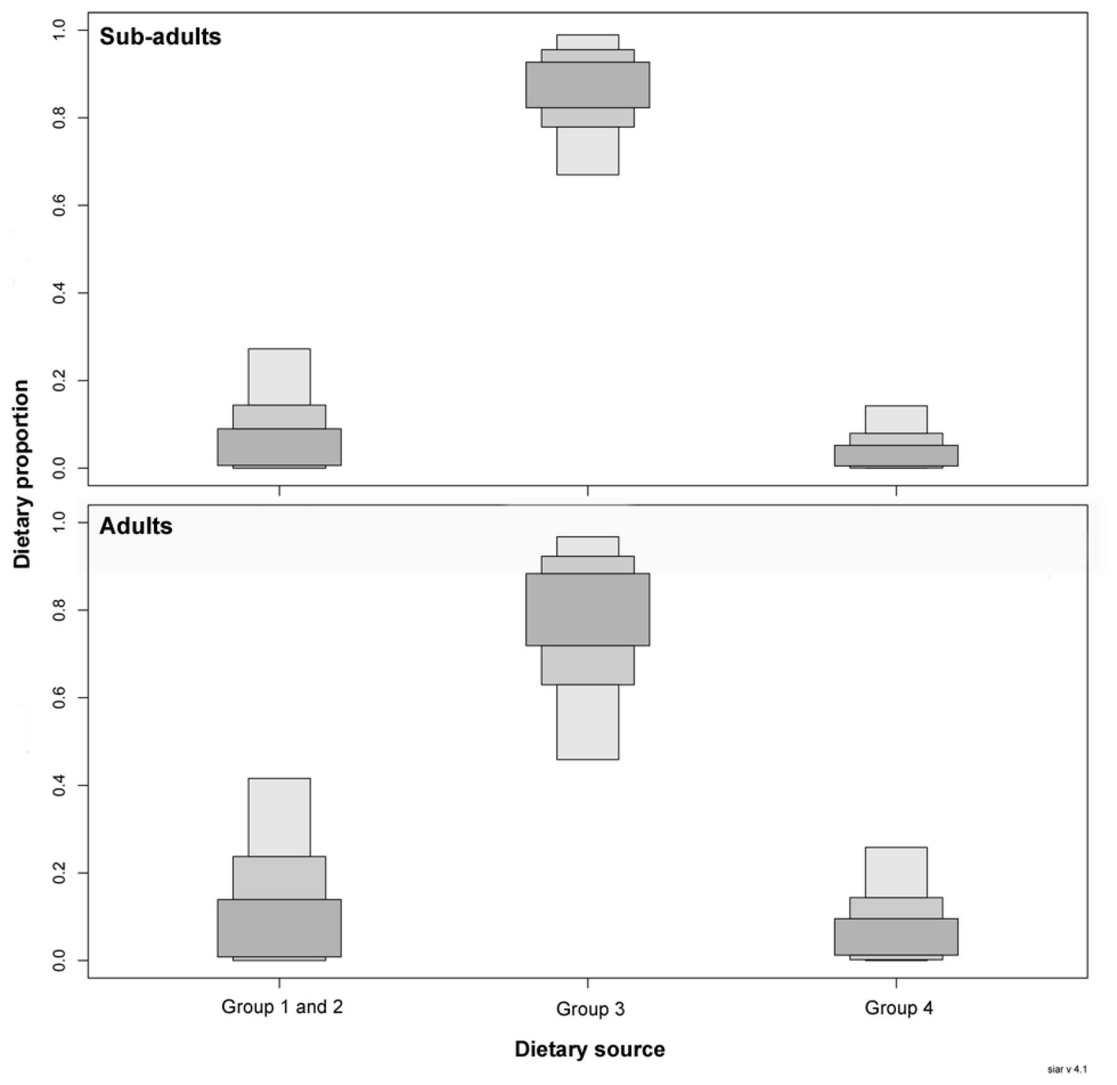
Boxplots from the SIAR mixing model showing the relative dietary contributions of potential prey sources (Groups 1-4) to the diet of sub-adult and adult bull sharks. Groups 1 and 2 were combined for the analysis due to similar stable isotopic signatures between these groups. The dietary proportions indicate the credibility intervals at 25, 75 and 95%.

The mixing model suggested that dietary items from group 3 make up the largest proportion of both sub-adult (84.2%) and adult (73.8%) bull shark diet (Figure 3). Sub-adult bull sharks had smaller contributions from combined groups 1 and 2 (10.1%) and group 4 (0.05%) relative to adult bull sharks that had greater contributions from combined groups 1 and 2 (16.0%) and group 4 (10.1%). Figure 3 indicates the credibility intervals at 25, 75 and 95% associated with these mean values. 

### Niche width

**Figure 4:**
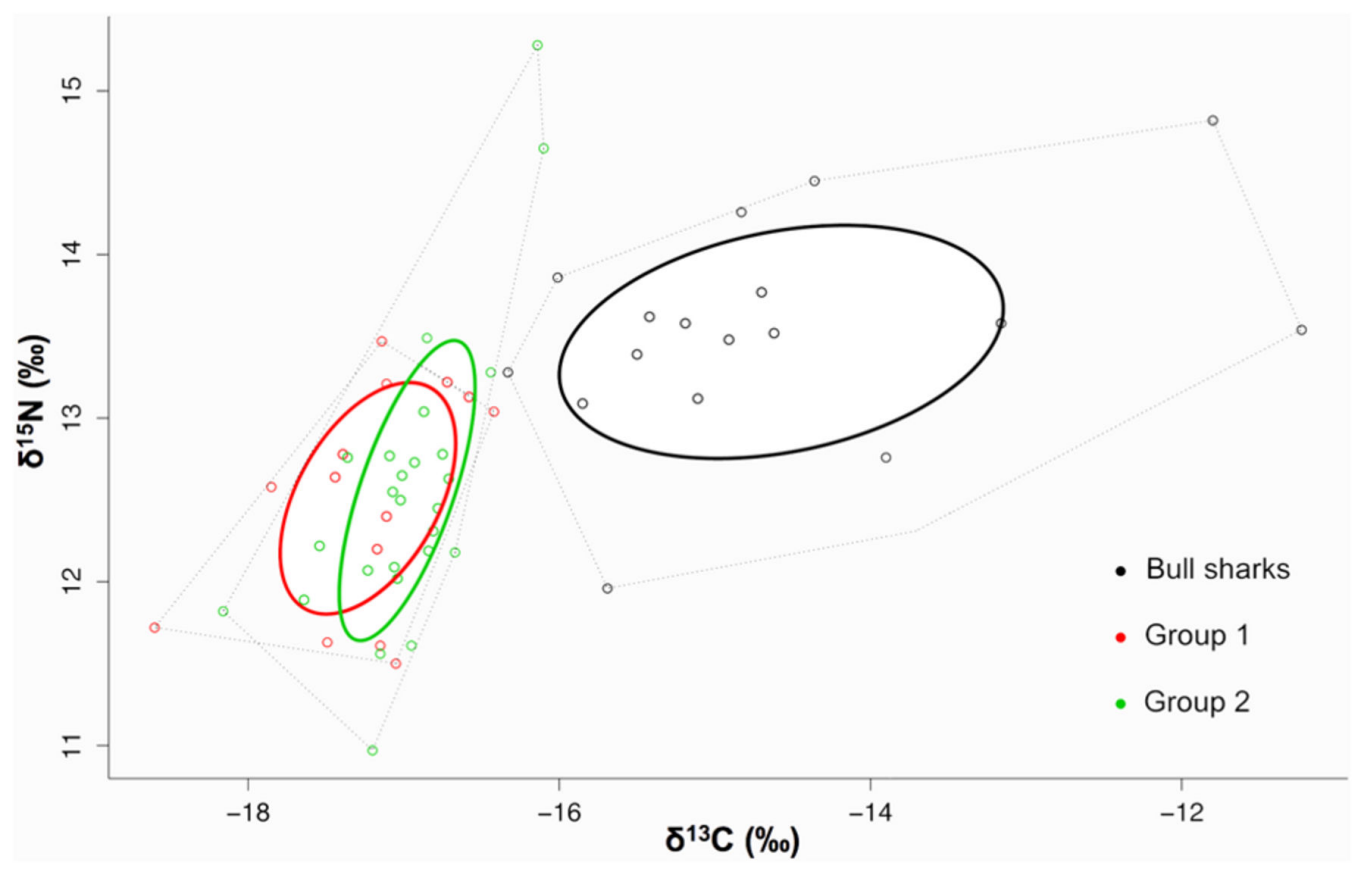
A dual isotope plot representing the niche widths of bull sharks (black line), fish group 1 (red line) and fish group 2 (green line). Calculated niche width is represented by the small sample size corrected ellipses (solid lines) and displayed in a δ^13^C-δ^15^N niche space.

Bayesian ellipse areas represent the niche widths of bull sharks and fish groups 1 and 2 in isotopic niche space (Figure 4). The niche width of bull sharks was significantly larger than both fish in group 1 (0.98 probability) and group 2 (0.99 probability). The variance of δ^13^C values exhibited by bull sharks was, in both cases, significantly greater than groups 1 (p<0.05) and 2 (p<0.05), accounting for the majority of the difference between niche widths. In contrast, the variance of δ^15^N values exhibited by bull sharks and fish groups 1 (p>0.05) and 2 (p>0.05) were not significantly different. There were no significant differences in niche width between group 1 and 2 (0.67 probability) and group 1 exhibited a 61% overlap with group 2 in niche space (Figure 4).

**Figure 5:**
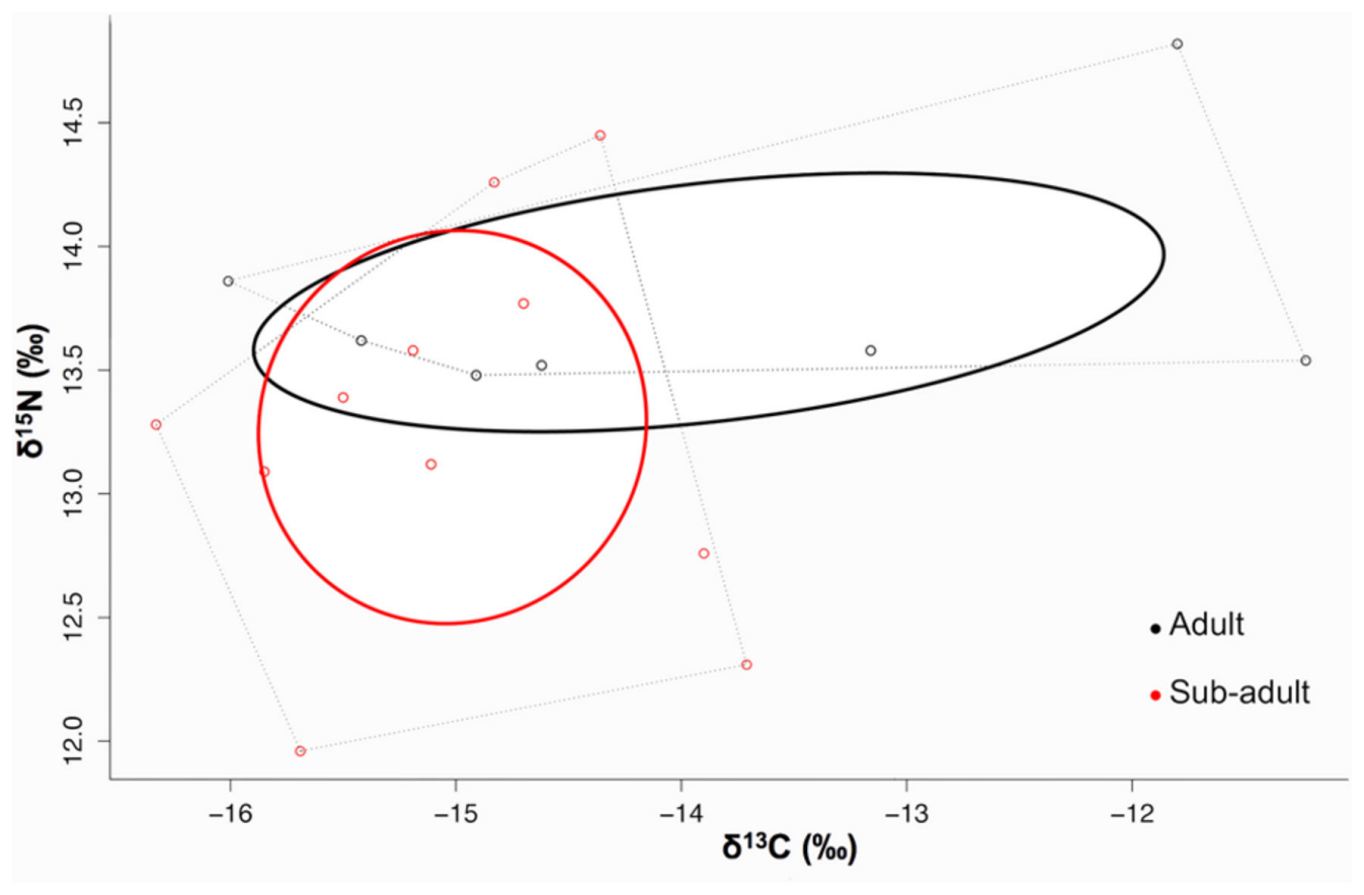
A dual isotope plot representing the niche widths of adult (black line) and sub-adult (red line) bull sharks. Calculated niche width is represented by the small sample size corrected ellipses (solid lines) and displayed in a δ^13^C-δ^15^N niche space.

The niche width of adult bull sharks (3.1) was greater than sub-adults (2.1) (0.85 probability) and exhibited a degree of niche overlap (adult niche width overlapped sub-adult niche width by 60%) (Figure 5). Neither the δ^13^C nor the δ^15^N values were significantly different (p>0.05 in both cases) between sub-adult and adult sharks. The variance of δ^13^C values was significantly different (p<0.05) but the variance of δ^15^N values was not significantly different (p>0.05) between sub-adult and adult sharks. 

### Trophic position

The mean calculated TP_SIA_ of bull sharks was 4.5 (±0.3). Adult sharks (4.6 ± 0.2, mean ± SD) exhibiting a higher TP_SIA_ compared with sub-adult bull sharks (4.4 ± 0.3, mean ± SD). 

## Discussion

### Community isotopic niche space

The range of δ^13^C values exhibited by all fish groups in the sampled community were within the range of baseline samples obtained from the same area by a previous investigation [58] (Table 2, Figure 2). The broad range of δ^13^C values exhibited by group 3 suggests that the various species within the group had a relatively varied diet as suggested by the literature [41-47]. In contrast, fish groups 1 and 2 had a more specialized diet with a similarly depleted and relatively narrow range of δ^13^C values, suggesting that species from groups 1 and 2 are foraging primarily on a δ^13^C depleted prey source, most likely consisting of small planktivorous fish species that are typically δ^13^C depleted relative to inshore sources [16,58]. *In situ* observations supported this and suggested a common association between fish species from group 1 and 2 and an abundance of small planktivorous fish species mainly from the family Caesionidae. The δ^13^C and δ^15^N values exhibited by elasmobranch group 4 were more enriched than fish groups 1 and 2 with a range of δ^13^C values more similar to those of the sampled bull shark population. Group 4, however, did have a relatively more enriched δ^15^N value range compared to bull sharks and this suggested a dietary contribution from prey with enriched δ^15^N signatures. Although bull sharks and species from group 4 may have had overlapping diets to some extent, it is likely that group 4 had a relatively higher contribution of a more enriched pelagic dietary source. This is supported by the literature [26] and *in situ* observations that suggest that to some extent the blacktip shark (*Carcharhinus limbatus*) in group 4 may prey on similar dietary items as bull sharks but they are typically more pelagic than bull sharks [26]. 

### Bull shark niche space

Bull sharks with isotopic values that diverged most from the values exhibited by the fish community at the study site appeared to have more enriched δ^13^C values implying that their prey originates from a more enriched δ^13^C community. Considering the biogeographic gradients along the southern African east coast suggested by Hill et al [58], these sharks most likely forage further south of the study site and / or primarily inshore. The isotopic values of some sharks were in fact similar to the coastal base source near Durban, South Africa [16] although it would be difficult to account for as other factors such as estuarine habitat use may have contributed to a change in isotopic ratios [59,60]. The relatively slow isotopic turnover rate of elasmobranch muscle tissue [13] may also mean that sharks foraging between different habitats will not reach isotopic equilibrium with one habitat which makes the interpretation of these data difficult. This may mean that the isotopic signatures reflected by these muscle tissues are in fact a mix of the dietary contributions from all frequented habitats [22] and may not reflect specific dietary contributions from the study site. However, these signatures may still provide a relative measure between individual bull sharks that reflect the differences between foraging ranges. Considering that bull sharks are capable of substantial movements [33,34] it is likely that a proportion of the population forages over a wide geographical range on the east coast of southern Africa. This is supported by records of bull sharks from the Eastern Cape coast [61] and the migration of a male adult bull shark from the Western Cape coast to the Mozambican coast over a distance of approximately 2000km (M. McCord pers. com). 

### Bull shark dietary contributions

The mixing model suggested that fish species from group 3 made up the largest proportion of potential bull shark diet within the sampled community. The relatively high contribution of similar mesopredatory fish species to the diet of large bull sharks is consistent with previous studies [31,59] but the relatively small contribution of elasmobranchs in the diet of adult sharks contrasts those sampled on the east coast of South Africa [31]. However, we expected differences in the dominant dietary items between the study site and the east coast of South Africa, as there is a steep biogeographical gradient along this coast resulting in substantial habitat differences between the study site and the lower east coast of South Africa. Although stable isotope analysis may not provide the taxonomic resolution of stomach content analysis, it does suggest that mesopredatory fish species do constitute a consistently important portion of bull sharks diet over time. Dietary contributions from top predatory fish species and shark species from groups 1, 2 and 4 were not as important as group 3, however, the mixing model suggested prey items from group 1, 2 and 4 made up a higher proportion of adult bull sharks diet. This is consistent with the increased ability of larger sharks to consume larger prey items from higher trophic levels, including other elasmobranch species [31,62]. Considering the diverse number of prey items that bull sharks are known to consume [31], it is possible that their prey spectrum at the study site may have been under represented. These may include other species of elasmobranchs that are known to constitute an important part of the prey of adult bull sharks caught in bather protection nets on the east coast of South Africa [31]. However, the snapshot nature of stomach content analysis probably reflected the recent foraging behavior of those sharks and may have been biased towards prey from near shore habitats where the bather protection nets are situated [31]. 

### Predatory niche width

Although top predatory fish assemblages (group 1 and 2) and bull sharks both had similarly high trophic positions, the significantly smaller niche widths of predatory fish assemblages represented by groups 1 and 2 relative to the sampled bull shark population suggest that bull sharks have a greater influence on their respective marine communities. Bull sharks exhibited a significantly larger range of δ^15^N values and δ^13^C values compared to the predatory fish assemblages suggesting that as a population they forage on a wider diversity of prey from various trophic levels. Although predatory teleosts are also capable of substantial migrations [63], bull sharks are more likely to forage amongst more diverse habitats, such as estuaries, and may exhibit individual foraging strategies [21]. Bull sharks are known to be generalist predators [24,31,59] that consume a variety of prey items but foraging behavior of individuals may be more complex. Matich et al [21] suggested that juvenile bull sharks exhibit a degree of dietary specialization individually but as a population, they consume a variety of prey items more typical of a generalist species. Foraging variability may also be influenced by the seasonal availability of resources and individuals or populations may adopt different foraging strategies according to changing environmental variables [64]. Data from this study were not sufficient to investigate individual specialization or diet variability, however, these studies [21,64] investigated juvenile sharks within the confines of an estuary and therefore, it is likely that the foraging strategies employed by adults would be different as they are more mobile, encounter a more diverse range of habitats and are not as constrained by predator risk effects [6]. In an environment where different food webs have a high degree of geographical overlap, predators may be more likely to utilize the most abundant food source regardless of the food web in which the resource occurs [65]. At the study site in this investigation, the pelagic and coastal food webs were not spatially distinct and bull sharks were easily able to move between them (pers obs.). Therefore, it is unlikely that individuals would exhibit foraging strategies limited to only one food web or resource pool. This would account for the broad range of δ^13^C values exhibited by bull sharks in this study and supports the argument that adult bull sharks are not dietary specialists albeit that foraging strategies of individuals may be complex [66] and deserve further investigation. 

### Adult vs sub-adult bull shark niche space

Due to challenges associated with sampling it was only possible to obtain a small sample size (n=9) from adult bull sharks. In order to account for this, the small sample size corrected Bayesian ellipse analysis was employed [54] which dealt well with the limited sample sizes. However, it is also acknowledged that typically sample sizes smaller than 10 can lead to increased variance in the Bayesian model output and may lead to an enlarged ellipse area estimate. Taking this into account, it was still apparent that adult sharks exhibited a shift in niche space towards a more enriched δ^15^N diet with a wider range of δ^13^C values. Evidence from the mixing model supports this hypothesis, suggesting that larger predatory fish species made a large contribution to the diet of adult sharks. The higher calculated trophic position of adult sharks also reflected this. Additionally, supporting evidence from the literature suggests that adult sharks consume a greater proportion of larger prey from higher trophic levels [31,59]. Adult sharks also exhibited a significantly wider range of δ^13^C values implying that they source their dietary items from a more diverse habitat range than sub-adults. These results were also consistent with the published literature that suggests larger sharks typically range over broader geographical areas [29,59,67]. This suggests that bull sharks not only undergo an expanded dietary range related to prey size [31] but may also exhibit a niche shift consistent with an expanded foraging area [68]. In many marine communities, the increased mobility of adults is an important mechanism through which spatially separated communities are connected, and ensures energy transfer that link ecological processes which may maintain the functionality of these systems [69]. Factors such as gender and individual foraging behavior may however require additional investigation in order to further elucidate the ecological role of bull shark populations. 

### Bull shark trophic position

The range of δ^15^N values used to calculate the trophic position (TP_SIA_) for bull sharks was similar to other studies that investigated large shark species [11,16,23,59]. The lack of an ontogenetic relationship between the size of bull sharks and δ^15^N was, however, in contrast to some of these studies [16,22,70]. This was most likely due to the limited size range of individuals sampled in this study and the absence of juveniles from the study site. The narrow range of δ^15^N values exhibited by adult bull sharks also suggests that these sharks are close to a δ^15^N plateau and that they all feed at a consistently high trophic level [19], consequently decreasing the size based variation in δ^15^N values. 

The mean TP_SIA_ for bull sharks (4.5) based on stomach content analysis alone (TP_SCA_) was slightly higher than the value of 4.3 reported by Cortes [9]. Although previous studies [16,71,72] reported a similar range of difference between TP_SIA_ and TP_SCA_ when calculating TP_SIA,_ Hussey et al [7] found that TP_SIA_ values typically are higher than TP_SCA_ values. Calculated TP_SIA_, however, can vary considerably depending on the assigned TP of the base consumer, the chosen fractionation rate and the type of tissue sampled [7,73]. Therefore, while the absolute value of calculated TP_SIA_ should be used with caution, the relative value calculated in this study provided a meaningful comparison between bull sharks and fish assemblages within the sampled community. As expected, bull sharks occupied a high trophic position within the sampled community consistent with the trophic positions of other large shark species calculated from stable isotope analysis [11,16]. 

## Conclusions

This study suggests that although top predatory teleost fish and large shark species may have similarly high trophic positions, their predatory role within their respective marine communities may be functionally different. A single large shark species such as the bull shark may exert a disproportionally large predatory influence within and amongst various marine communities due to their ability to prey on a broad diversity of prey items over a wide geographical range. The predatory influence of adult sharks may be particularly important as they increasingly consume prey from higher trophic levels and from a greater foraging range. Unlike predatory teleost fish assemblages, large shark species may be able to link important ecological processes within and between a diverse range of marine habitats. However, these processes may be complex and a further understanding of interpopulation and interindividual variability will elucidate the roles of such large shark species further. Bull sharks are slow growing and late maturing and thus have an intrinsic vulnerability to population decline [36,74] typically driven by fishing pressure [2,3,5] and habitat degradation [67,75]. Therefore, understanding how the spatial and temporal scales of these threats are linked to the variable feeding ecology of shark populations are important conservation management considerations. Ultimately this study highlights the need to ensure that conservation measures take into account the importance of large and mobile marine apex predators such as the bull shark. 
